# Sterile Testis Complementation with Spermatogonial Lines Restores Fertility to *DAZL*-Deficient Rats and Maximizes Donor Germline Transmission

**DOI:** 10.1371/journal.pone.0006308

**Published:** 2009-07-21

**Authors:** Timothy E. Richardson, Karen M. Chapman, Christina Tenenhaus Dann, Robert E. Hammer, F. Kent Hamra

**Affiliations:** 1 Department of Chemistry, University of Indiana, Bloomington, Indiana, United States of America; 2 Department of Biochemistry, University of Texas Southwestern Medical Center, Dallas, Texas, United States of America; 3 Department of Pharmacology, University of Texas Southwestern Medical Center, Dallas, Texas, United States of America; 4 The Cecil H. & Ida Green Center for Reproductive Biology Sciences, University of Texas Southwestern Medical Center, Dallas, Texas, United States of America; The University of Hong Kong, China

## Abstract

Despite remarkable advances in assisted reproductive capabilities ∼4% of all couples remain involuntarily infertile. In almost half of these cases, a lack of conception can in some measure be attributed to the male partner, wherein *de novo* Y-chromosomal deletions of sperm-specific *Deleted-in-Azoospermia* (*DAZ*) genes are particularly prevalent. In the current study, long-term cultures of rat spermatogonial stem cells were evaluated after cryo-storage for their potential to restore fertility to rats deficient in the *DAZ-like* (*DAZL*) gene. Detailed histological analysis of *DAZL*-deficient rat testes revealed an apparently intact spermatogonial stem cell compartment, but clear failure to produce mature haploid gametes resulting in infertility. After proliferating >1 million-fold in cell number during culture post-thaw, as few as 50,000 donor spermatogonia transplanted into only a single testis/recipient effectively restored fecundity to *DAZL*-deficient rats, yielding 100% germline transmission to progeny by natural mating. Based on these results, the potency and efficacy of this donor stem cell line for restoring fertility to azoospermic rodents is currently unprecedented. Prospectively, similar successes in humans could be directly linked to the feasibility of obtaining enough fully functional spermatogonial stem cells from minimal testis biopsies to be therapeutically effective. Thus, regeneration of sperm production in this sterile recipient provides an advanced pre-clinical model for optimizing the efficacy of stem cell therapies to cure a paradoxically increasing number of azoospermic men. This includes males that are rendered infertile by cancer therapies, specific types of endocrine or developmental defects, and germline-specific *de novo* mutations; all of whom may harbor healthy sources of their own spermatogonial stem cells for treatment.

## Introduction

In total, >5% of the male population is infertile, with male factors being attributed to nearly 50% of all infertile couples [1], [2]. On a global scale, >1% of all males are inflicted with a severe defect in sperm production termed azoospermia [3], [4], [5]. Fundamentally, because azoospermia results in an inability to reproduce by natural mating, it seems enigmatic as to why this disease remains so prevalent in the human population. Such an epidemiological trend clearly points to the existence of potent environmental factors that disrupt the process of sperm production (i.e. spermatogenesis) [4], [6], or a substantial repertoire of gene mutations that could render one sterile, but otherwise healthy [7], [8]. This is in fact true in mammalian species where mutations in >100 distinct genes have already been shown to disrupt sperm development or function in mice [7], [8]. In humans, ∼10% of males with non-obstructive azoospermia suffer from a ploidy defect termed Klinefelter Syndrome, where ∼1/750 boys are born with a 47, XXY karyotype [9], [10]. Additionally, a variety of micro-deletions from the Y-chromosome are particularly prevalent and have been linked to ∼20% of all cases of male-factor infertility [3], [10], [11], [12]. A majority these Y-chromosome microdeletions encompass genes in the DAZ family [12], [13].

Rodent genomes do not encode DAZ genes on their Y-chromosome, but do contain a highly conserved autosome, DAZL, that is specifically expressed in the germline [14], [15]. Like DAZ genes in humans, DAZL is required for gametogenesis in rodents [16], [17], [18]. In mice, DAZL function has been linked to maintenance of pluripotency [19], epigenetic programming in the embryonic germline [19], primordial germ cell/gonocyte survival [18], and is required for oogenesis and spermatogenesis [16]. In a new transgenic rat model, expression of DAZL was disrupted by forced expression of a complementary small hairpin RNA (shRNA) [17]. Interestingly, expression of DAZL in these “knockdown” rats remained sufficient to support fertility in females [17]. However, as with many men diagnosed with *DAZ* gene deletions, *DAZL*-deficient rats were sterile due to a severe block in spermatogenesis [17].

In addition to gene mutations that specifically disrupt germ cell function, another prominent concern resides in the fact that each year an increasing number of young women and men are left infertile due to the potent, toxic side-effects of life-saving cancer therapies on gametogenesis [6], [20], [21], [22]. It is estimated that ∼30% of male childhood cancer survivors are inflicted with azoospermia due to degenerative side effects of chemotherapy on spermatogenesis [21]. Bilateral cryptorchidism, which is the lack of testicular descent after birth, is another major cause of azoospermia in about 1/500 males born [5]. Thus, the best hope for these boys to someday father their own children currently depends on the cryopreservation of their spermatogonial stem cells. Still, this is under anticipation that cellular therapies for curing spermatogenic arrest are established as a clinical option early enough in their future.

As a new hope for many such men with azoospermia, a pioneering breakthrough in reproductive biology was the discovery that spermatogonial stem cells isolated from mouse testes could maintain their ability to generate fully functional sperm following transplantation into testes of another mouse [23], [24], [25]. Similar experiments soon followed with rats [26], and isolated mouse spermatogonia were next shown to maintain their regenerative potential after months in culture [27]. New culture media supporting the long term proliferation of rodent spermatogonial lines *in vitro* have since been formulated [28], [29], [30], [31], and scientists are now on the brink of establishing conditions required to cultivate human spermatogonial lines from testis biopsies as a key step toward using germline stem cells in regenerative medicine [32], [33].

Ostensibly, the ability to propagate human spermatogonial lines in culture, prior to using them to produce functional spermatozoa by transplanting them back into the testes of their own donor, presents a clear strategy to cure many existing types of male infertility. Establishing these culture methods would overcome the foreseeable barrier of obtaining enough pure, donor spermatogonia from a minimally invasive testis biopsy to effectively restore a patient's fertility. To date, a majority of studies in this area have been performed in mice [23], [25], [28], [29]. However, due in large part to the multipotent nature of isolated germline stem cells in culture [32], [33], [34], [35], and the potential for introducing defective cells back into patients, it will be informative to evaluate therapeutic efficacies of spermatogonia in additional pre-clinical mammalian models, as presented herein, using azoospermic *DAZL*-deficient rats [17].

## Results

### DAZL-Deficient Rats Maintain an Intact Sperm Stem Cell Compartment

**Figure 1:**
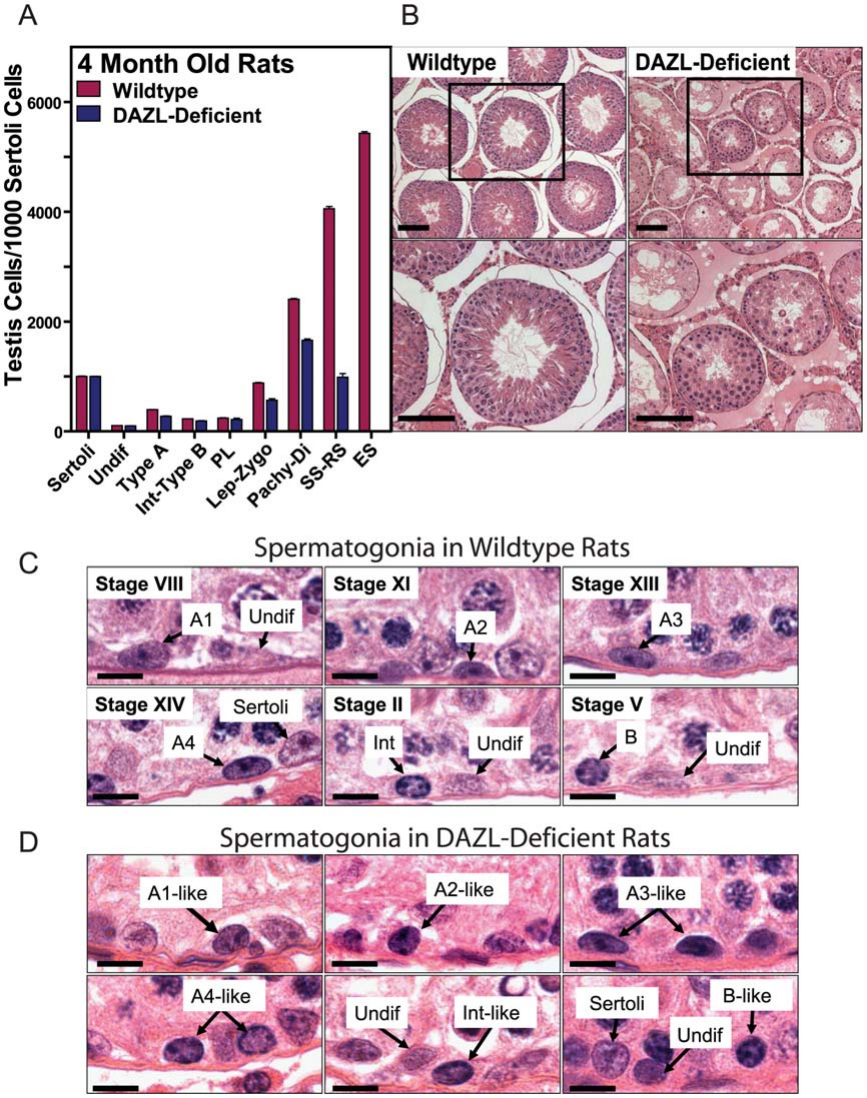
Analysis of Spermatogenic Failure in DAZL-Deficient Rats. (A) Relative numbers of spermatogenic cell types/1000 Sertoli cells in testes of 4 month old wildtype and transgenic *DAZL*-deficient rats (+/−SEM, n = 3 rats/group). Undifferentiated type A spermatogonia (Undif), differentiating type A spermatogonia (Type A), intermediate to type B spermatogonia (Int-Type B), preleptotene spermatocytes (PL), leptotene-zygotene spermatocytes (lept-zygo), pachytene-diplotene spermatocytes (Pachy-Di), secondary spermatocytes-round spermatids (SS-RS), elongating spermatids (ES). Significant differences (i.e. p<0.05) in testis cell types scored in wildtype and DAZL-deficient rats were determined for SS-RS (p = 0.022) and ES (p<0.0001) using unpaired two-tailed students t-test. (B) Histological cross-sections of seminiferous tubules from 4 month old wildtype (*Top left*) and transgenic *DAZL*-deficient rats (*Top right*). *Bottom left and right* show higher magnification images of boxed regions in the *Top* panels. Scale bars = 100 µm. (C) Images of spermatogonial types in testis sections from wildtype rats at stages VIII, XI, XIII, XIV, II and V of spermatogenesis. Types of undifferentiated (Undif) and differentiating spermatogonia (A1, A2, A3, A4, Int, B) were determined by analysis of staining patterns in nuclei at specific stages of spermatogenesis. Scale bar = 30 µm. (D) Images of spermatogonial types in testis sections from *DAZL*-deficient rats. Because stages of the epithelial cycle could not be classified in these rats, types of undifferentiated (Undif) and differentiating spermatogonia (A1-like, A2-like, A3-like, A4-like, Int-like, B-like) were estimated based on staining profiles of spermatogonia in wildtype rats. Scale bar = 30 µm.

**Figure 2:**
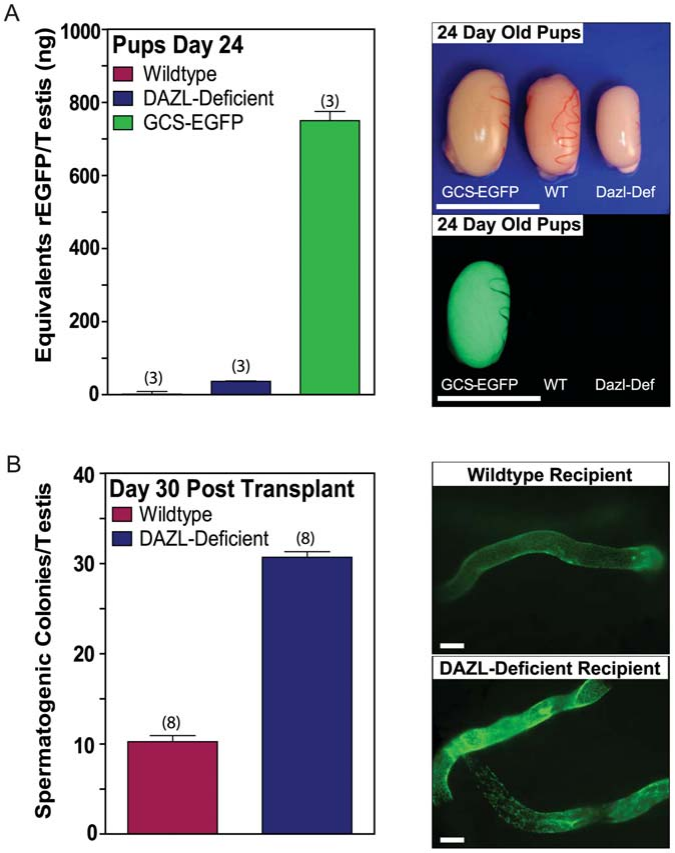
DAZL-Deficient Rats are Efficient Spermatogonial Recipients. (A) Relative abundance of EGFP in testes of Wildtype, *DAZL*-deficient and *GCS-EGFP* rats. *Left*: Data expressed as the equivalents of recombinant, histidine-tagged EGFP (rEGFP)/testis (+/−SEM, n = 3 testes/rat strain) as determined by fluorometry of testis extracts at 24 days of age. *Right*: Bright field (top) and green fluorescence (bottom) images of testes dissected from *GCS-EGFP*, wildtype (WT) and *DAZL*-deficient (Dazl-Def) rats at 24 days of age. Scale bar = 1 cm. (B) Spermatogenesis colony forming assays using *DAZL*-deficient rats as recipients. *Left*: Numbers of spermatogenic colonies formed/testis by donor *GCS-EGFP* rat spermatogonia in Wildtype (10.25+/−0.68 colonies/testis, +/−SEM, n = 8 testes) and *DAZL*-Deficient rats (30.69+/−0.62 colonies/testes, +/−SEM, n = 8 testes) at 30 days following transplantation; p<0.0001, unpaired two-tailed students t-test. Donor spermatogonia were transplanted at passages 15 and 17 (i.e. culture days 182 and 204) at 2000 *GCS-EGFP* ^+^ cells/testis. *Right*: Images of individual colonies of spermatogenesis in Wildtype and *DAZL*-deficient recipient rats that were generated by the donor *GCS-EGFP* spermatogonia (green fluorescence is from donor cells). Images are representative of colonies scored and plotted in the *Left* panel. Scale bar = 100 µm.

Here, we tested the hypothesis that following cryopreservation, long term cultures of proliferating spermatogonia could be used to restore fertility to *DAZL*-deficient rats [17]. Detailed morphometric analysis of the spermatogenic defect in *DAZL*-deficient rats revealed normal numbers of spermatogonia, but reduced numbers of spermatocytes and round spermatids in their seminiferous tubules, culminating in a severe failure to produce elongating spermatids (Fig. 1A, B). However, based on these analyses, *DAZL*-deficient rats appeared to express an intact spermatogonial stem cell compartment, as evidenced by both undifferentiated and differentiating populations of spermatogonia within seminiferous tubules of adult animals (Fig. 1C, D). The presence of a functional spermatogonial stem cell compartment was verified by *in vivo* spermatogenesis colony forming assays in which genetically tagged donor spermatogonia were thawed from cryo-storage, propagated over multiple passages in culture, and then transplanted into seminiferous tubules of busulfan-treated, *DAZL*-deficient rat testes (Fig. 2). Donor spermatogonial lines were derived from testes of individual *Germ Cell Specific* (*GCS*)*-EGFP* transgenic rats [31], [36]. *GCS-EGFP* rats robustly expressed EGFP as a vital marker specifically during all known steps of gametogenesis [37]. The *DAZL*-deficient recipient rat line also expresses EGFP from its transgene, but at relatively low levels [17]. By comparison, EGFP is 20-fold more abundant in testes of *GCS-EGFP* rats than in *DAZL*-deficient rats (Fig. 2A). Thus, development of donor spermatogonia from *GCS-EGFP* rats was clearly detected following transplantation into *DAZL*-deficient rat testes, which they colonized 3-fold more efficiently than wildtype recipient testes (Fig. 2B).

### Donor Sperm Stem Cells Effectively Restore Fertility to Dazl-Deficient Rats

**Figure 3:**
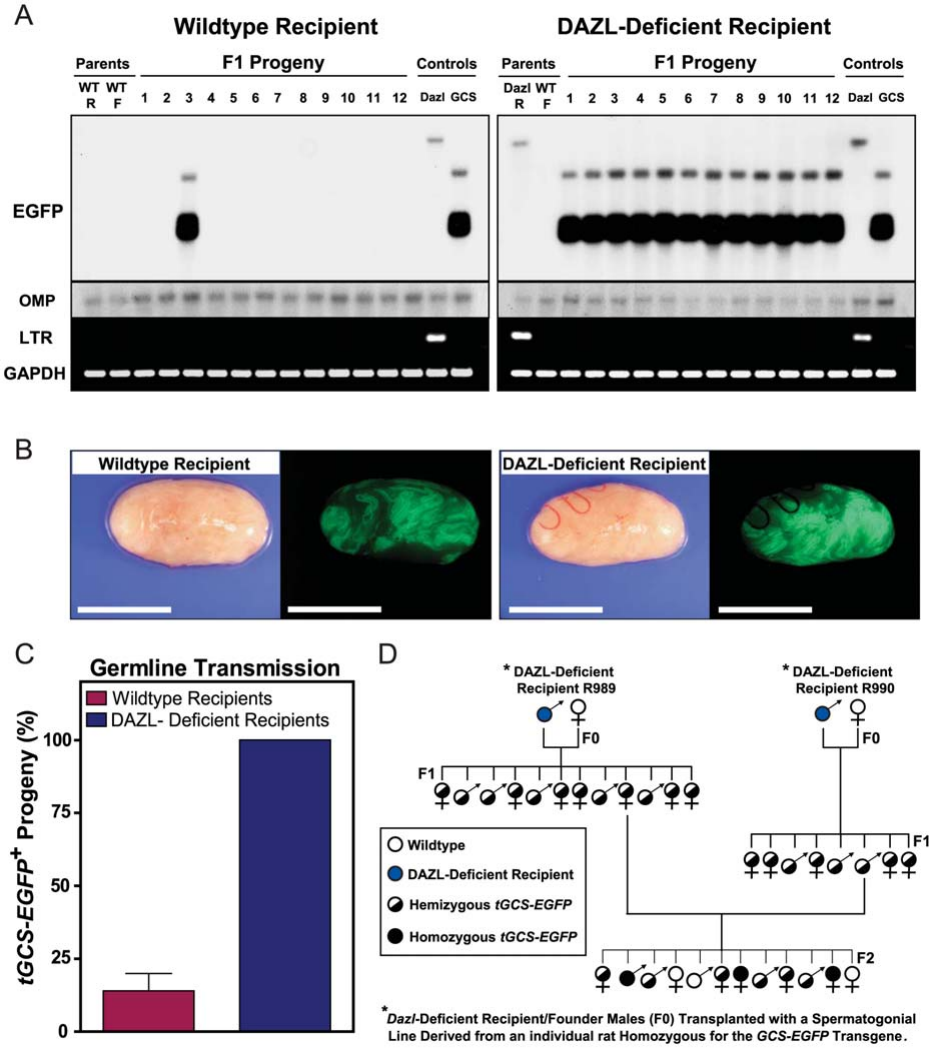
Maximal Donor Germline Transmission from DAZL-Deficient Rats. (A) Southern blot analysis of progeny from Wildtype (WT) and DAZL-Deficient (Dazl) recipient rats transplanted with 50,000 *GCS-EGFP* spermatogonia/right testis at passage 13 (i.e. 158 days in culture); left testes of each animal were not transplanted. At 75 days post-transplantation recipients (R) were paired with WT females (F) and allowed to produce pups by natural breeding (See R942-R949 in Table 1). Shown are blots from representative litters probed for *EGFP* to distinguish progeny produced by donor cells. OMP = loading control. Genomic DNA Controls were from untreated *GCS-EGFP* and *DAZL*-Deficient transgenic rats. LTR = PCR primers specific for lentiviral transgene in *DAZL*-deficient rats. GAPDH = PCR loading control. (B) Bright field and green fluorescence images of testes from Wildtype (*Left*) and DAZL-deficient (*Right*) recipient rats at 212 days post-transplantation. Scale bar = 1 cm. (C) Graph of germline transmission rates for the donor, *GCS-EGFP* transgene from Wildtype and DAZL-deficient recipient rats transplanted with 50,000 *GCS-EGFP* spermatogonia/right testis at passage 13; left testes were not transplanted. *DAZL*-deficient recipients transmitted the *GCS-EGFP* transgene to 100%+/−0% of progeny (+/−SEM, n = 3 recipients; 9 litters), with 73 of 73 total F1 pups born from donor cells. Wildtype recipients transmitted the *GCS-EGFP* transgene to 14%+/−5.9% of progeny (+/−SEM, n = 3 recipients; 9 litters), with 16 of 116 total F1 pups born from donor cells. (D) Genealogy tree showing stable transmission of donor haplotypes from *DAZL*-Deficient recipients (F0) R989 and R990 to F1 and F2 progeny. Recipients were each transplanted with 150,000 rat spermatogonia/testis from line RSGL-GCS9 at passage 17 (See R988-R990 in Table 1). Spermatogonial line RSGL-GCS9 was derived from a rat homozygous for the *GCS-EGFP* transgene [36]. Thus, F1 progeny represent half-siblings; some of which were crossed to re-derive transgenic F2 progeny homozygous for the *tgGCS-EGFP* allele.

**Figure 4:**
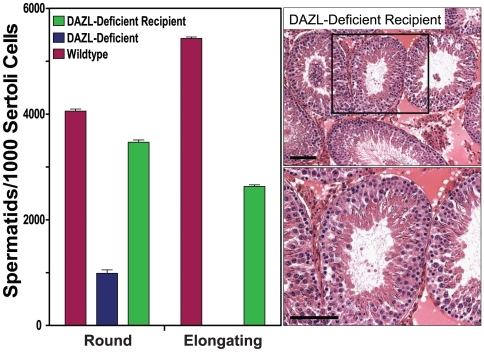
Long-Term Spermatogenic Potential of Donor Spermatogonia. (*Left*) Graph showing relative numbers of Round and Elongating Spermatids in seminiferous tubules of non-transplanted, non-busulfan-treated, Wildtype and *DAZL*-deficient rat lines at 4 months of age (see Fig. 1a), in comparison to Spermatid numbers in busulfan-treated, *DAZL*-deficient recipient rats at 212 days (i.e. ∼8 months of age) after being transplanted with rat spermatogonial line, RSGL-GCS9, at passage 13 (i.e. culture day 158). Cell counts were normalized/1000 Sertoli cells. +/−SEM, n = 3 rats/group. (*Right*) Images of histological sections of seminiferous tubules from the *DAZL*-deficient recipient rats described in the “Left” panel after being transplanted with spermatogonia from RSGL-GCS9. *Bottom Right* shows a higher magnification image within the boxed region of the *Top Right* panel. Scale bars = 100 µm.

The transplanted spermatogonial stem cells effectively developed into functional spermatozoa, which due to the absence of sperm competition, transmitted the donor cell haplotype to progeny >7-fold more efficiently from *DAZL*-deficient recipients than from wildtype litter mates (Fig. 3A–C; Table 1). In each *DAZL*-deficient recipient, spermatogenesis was regenerated from the spermatogonial lines that had proliferated in culture (>2 million-fold expansion in cell number) for 5–7 months, yielding 100% germline transmission of the donor haplotype to F1 progeny by natural mating (Fig. 3A–C; Table 1). The *GCS-EGFP* transgene was further transmitted at Mendelian ratios from F1 to F2 progeny (26% wildtype, 48% heterozygous, 26% homozygous; 81 total pups; n = 6 litters) (Fig. 3D). No evidence of tumor formation was observed in any of the recipients or progeny. The regenerative effects of the spermatogonial lines on fertility were also apparent upon histological examination of testes from *DAZL*-deficient recipients at 212 days following transplantation; 57±6.1% (±SE, n = 3) of their seminiferous tubules contained colonies of spermatogenesis that developed to the elongating spermatid stage (Fig. 4). This represented a >2000-fold increase in the relative number of elongating spermatids scored/number of Sertoli cells in transplanted versus non-transplanted *DAZL*-deficient rat testes (Fig. 4).

**Table 1:** Progeny from Wildtype and *DAZL*-Deficient Recipient Rats Transplanted with GCS-EGFP Rat Spermatogonia.

Recipient	Recipient Background	Culture Passage Number	Days to Analysis	Gram wt per R, L testis	Cells per R, L testis	Days to first litter^1^	Number Litters	Average Litter Size	Total Pups Born	Pups Born from Transplanted Stem Cells^2^ (%)^3^
**Group 1**										
R946	Wildtype	p13	212	0.569, 0.341	50,000∶0	127	3	11.3	34	3 (8.8)
R948	Wildtype	p13	212	0.735, 0.278	50,000∶0	131	3	10	30	11 (36.7)
R949	Wildtype	p13	212	1.054, 0.201	50,000∶0	113	3	17.3	52	2 (3.8)
Average		p13	212	0.786, 0.273	50,000∶0	123.7	3	12.9	38.7	4.3 (16.4)
**Group 2**										
R942	DAZL-Def	p13	212	0.572, 0.286	50,000∶0	154	3	8.7	26	26 (100)
R943	DAZL-Def	p13	212	0.651, 0.377	50,000∶0	141	3	7.7	23	23 (100)
R944	DAZL-Def	p13	212	0.511, 0.326	50,000∶0	156	3	8	24	24 (100)
Average		p13	212	0.578, 0.330	50,000∶0	150	3	8.1	24.3	24.3 (100)
**Group 3**										
R988	DAZL-Def	p17	236	0.523, 0.578	150,000∶150,000	122	3	12.7	38	38 (100)
R989	DAZL-Def	p17	236	0.489, 0.439	150,000∶150,000	128	3	9.3	28	28 (100)
R990	DAZL-Def	p17	236	0.487, 0.371	150,000∶150,000	136	3	5.3	16	16 (100)
Average		p17	236	0.499, 0.427	150,000∶150,000	128.6	3	9.1	27.3	27.3 (100)

Wildtype or DAZL-deficient (DAZL-def) recipient rats were transplanted with either 0.5 or 1.5×10^5^ EGFP^+^ cells/testis from rat spermatogonial line GCS9 at 12 days after busulfan treatment (i.e. 12 mg/kg i.p.) on postnatal day 24. At ∼75 days post-transplantation recipients were paired with 75–80 day old wildtype female rats. Spermatogonia line GCS9 was harvested from passages number 13 and 17, which corresponded to 158 and 204 days in culture, respectively, prior to their transplantation. Recipients R942-R949 were littermates born from a hemizygous, transgenic DAZL-deficient female and a wildtype Sprague Dawley male. No progeny were born from breeder pairs of un-transplanted, busulfan-treated DAZL-deficient males and wild-type females (n = 3 breeder pairs). Breeder pairs of untreated, wild-type male litter mates of DAZL-deficient rats and wild-type female rats from Harlan, Inc. produced 15.5±4.5 pups/litter (+/−SEM, n = 8 litters from 3 breeder pairs).

1 p = 0.0209 Average Group 1 versus Average Group 2; p = 0.0251 Average Group 2 versus Average Group 3.

2 p = 0.0031 Average Group 1 versus Average Group 2.

3 Percent *GCS-EGFP* ^+^ F1 progeny.

## Discussion

Defining molecular mechanisms linked to the enhanced colonization of donor spermatogonia in *DAZL*-deficient recipients will be of potential clinical interest because it could lead to new therapeutic strategies for optimizing the efficiency at which donor germ cells engraft testes of infertile men (*See* *Fig. 2B*). The ability to apply such knowledge would theoretically reduce the number of donor stem cells required to successfully restore a patient's fertility. In specific cases, reducing the number of donor stem cells required per procedure would directly reduce the size of testicular biopsies needed from the patient. Accordingly, reducing the number of stems cells needed from a given biopsy would also directly decrease the relative amount of time for *in vitro* expansion of a donor spermatogonial line to obtain enough cells in a pure form to preserve fertility. In turn, the ability to shorten the time required for efficient *in vitro* expansion of donor cells using smaller amounts of testicular tissue could prove to be critical for cancer survivors that bank their spermatogonial stem cells prior to therapy.

In addition to colonizing *DAZL*-deficient rat testes more efficiently, donor spermatogonial lines transmitted their haplotypes from *DAZL*-deficient recipients more efficiently. Depending on the case at hand, it would also be important to anticipate the relative levels of sperm competition that could occur between a patient's donor and non-transplanted, endogenous germlines in response to currently unexplored therapeutic factors linked to autologous transplantation protocols. Biologically, this would apply to situations in germline mosaics where genetic modifiers uniquely expressed by donor spermatogonia could potentially rescue the development of endogenous spermatogonial lines that harbor undesirable genetic traits. However, in the current model, no *DAZL*-deficient progeny were born from transplanted *DAZL*-deficient rats (0/155 total F1 pups) exposed to healthy donor spermatogonia. Thus, neither down-stream effectors of *DAZL* produced by the wildtype donor cells, nor other procedural effects of the transplant were sufficient to stimulate development of functional spermatozoa from *DAZL*-deficient rats to levels readily transmittable by natural breeding. These genetic data support the hypothesis that germline-specific expression of DAZL is sufficient to fully rescue male fertility in DAZL-deficient mammals.

Initial studies in monkeys demonstrate the feasibility of the autologous transplantation approach in primates [38], [39], [40]. However, more definitive quantitative studies on the ability of genetically tagged spermatogonial stem cells to colonize, proliferate and undergo spermatogenic development after autologous transplantation into non-human primates await [20], [22]. Moreover, numbers of functional spermatogonial stem cells that can be isolated/gram weight of monkey or human tissue have yet to be clearly estimated. Due to the relative cost and technical demand associated with transplantation experiments in non-human primates, studies aimed at defining molecular mechanisms with potential to help optimize the efficacy of donor spermatogonia are being applied primarily in rodent models [41]. For example, progressive studies in rat and mouse models for cancer therapy have established that colonization and development of donor spermatogonia in irradiated testes can be enhanced by pharmacological suppression of testosterone [41], [42], and that transplantation of Sertoli cells can stimulate development of spermatogonia in rat testes damaged by irradiation [43]. These experimental designs in rodents could now be translated to non-human primates [20], [22].

Currently, many infertile men are unable to produce spermatozoa (i.e. azoospermia), or have low sperm counts (i.e. oligospermia) due to *de novo* deletion of one to four members in the *DAZ* gene family from the human Y-chromosome [12], [13]. Variants of these microdeletions account for ∼18% of men with non-obstructive azoospermia or severe oligospermia [44]. Rodent genomes do not have *DAZ* genes on their Y-chromosome, but do express a single homologue of human *DAZL* from chromosome 17 specifically in the germline [14]. Consistent with the phenotype in *DAZL*-deficient rats, spermatogenesis defects in *DAZL*-deficient mice mirror many forms of male-factor infertility caused by Y-chromosome microdeletions of human *DAZ* genes. Like in humans [12], the severity of spermatogenic failure in these mutant rodents ranges widely from a Sertoli-cell-only phenotype [18] to pre-meiotic [45] and meiotic [17], [46] stages of spermatogenic arrest; these phenotypes highlight the potential impact of yet to be defined genetic modifiers on spermatogenesis. Moreover, a recent report by Haston, *et al.*, provides new insight into the potential etiology of these defects by showing that DAZL is required to maintain proper epigenetic and genetic programs at multiple stages during gametogenesis [19].

**Figure 5:**
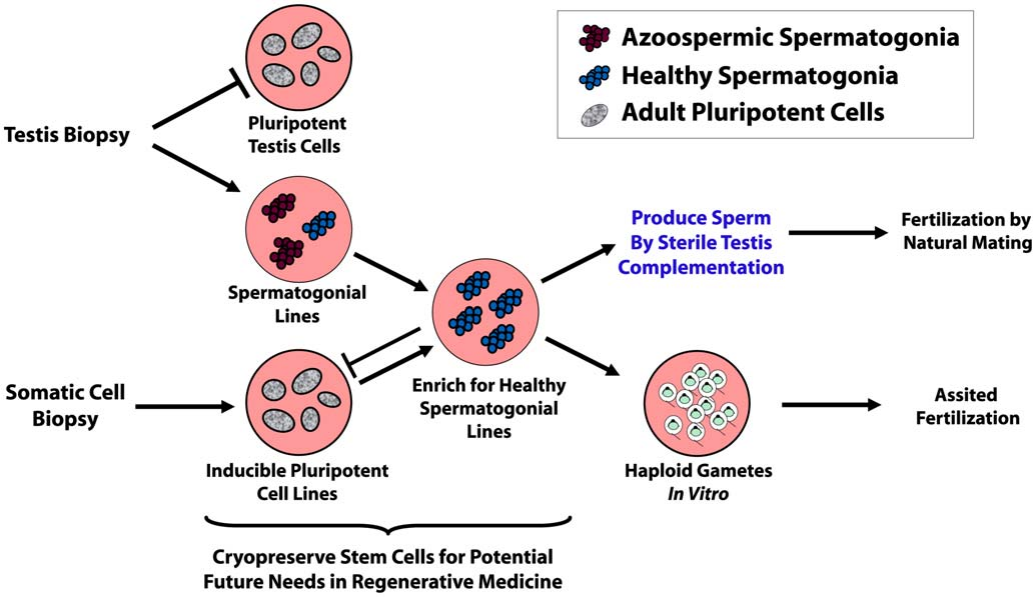
Prospective Procedures for Curing Azoospermia with Stem Cells. In many cases of spermatogenic failure, healthy sources of spermatogonia could be obtained from either a biopsy of an azoospermic patient's own testis, or prospectively, from a biopsy of his own somatic cells following induction into a pluripotent state. Pure spermatogonial lines derived from either source would require that they are maintained in a spermatogenic lineage and prevented from acquiring a pluripotent state when in culture. If such protocols were established, naturally healthy spermatogonial lines could be selected for from a background of potentially unhealthy cell lines, such as from cancer patients, or azoospermic men diagnosed as germline mosaics (later example is shown). Once selected for, the healthy lines could then be induced to develop into sperm by transplanting them back into the patient's own testes. As potential alternatives to sterile-testis complementation that are currently being investigated, spermatogonial stem cells could be induced to develop through meiosis during culture *in vitro*, or within tissue grafts for production of haploid gametes [50]. The resulting haploid spermatids could then be used for assisted fertilization.

Given extraordinary advances in regenerative medicine, together with the advent of spermatogonial culture [27], [28], [31], [36], clinical application of stem cells to cure specific types of infertility now appears more promising than ever (Fig. 5). Once optimized for specific curable cases of azoospermia, the ability to regenerate spermatogenesis using proliferating cultures of human spermatogonial stem cells holds remarkable potential to not only help battle infertility as a disease, but as a condition that can have profound, detrimental psychological effects on the quality of people's lives whom cannot parent their own children [47]. Defining molecular mechanisms that contribute to the enhanced efficacy of donor spermatogonia in *DAZL*-deficient rats could open new doors to advance this endeavor.

## Materials and Methods

### Animal Care and Use

Protocols for the use of rats in this study were approved by the Institutional Animal Care and Use Committee (IACUC) at UT-Southwestern Medical Center in Dallas. Rats used for this study were housed in individually ventilated, Lab Products 2100 cages in a dedicated room with atmosphere controls set to 72°F, 45–50% humidity during a 12 hour light/dark cycle (i.e. Light cycle = 6:00am-6:00pm, Central Standard Time adjusted for daylight savings time). Rats were fed Harlan Teklad Irradiated 7912, LM-485 Mouse/Rat Diet, 5% fat Diet and a continuous supply of reverse osmosis water.

### Rat Spermatogonial Lines

Seminiferous tubules were isolated from testes of 23–24 day old homozygous *SD-Tg(ROSA-EGFP)2-4Reh* Sprague Dawley rats. Rats of the *SD-Tg(ROSA-EGFP)2-4Reh* line were produced by pronuclear injection, exhibit germ cell-specific expression of enhanced green fluorescent protein (EGFP) throughout male and female gametogenesis, and are referred to as *GCS-EGFP* rats [37]. For deriving each spermatogonial line, seminiferous tubules were isolated from an individual rat and then enzymatically and mechanically dissociated into a cellular suspension to generate a testis cell culture [48]. The testis cell culture was then used to isolate enriched populations of laminin-binding germ cells, which are highly enriched in spermatogonial stem cells [48]. The laminin-binding fraction of germ cells was then used to derive proliferating cultures of spermatogonia [i.e. rat spermatogonial lines RSGL-GCS9 and RSGL-GCS10] in Spermatogonial Culture Medium (SG Medium) [36]. Passage 11, RSGL-GCS9 was thawed after 396 days of cryo-storage at −196°C in SG Medium containing 10% DMSO (i.e. Spermatogonial Freezing Medium), and then sub-cultured in fresh SG Medium, as previously described [36], for use in experiments between passage 13–17.

### Germ Cell Transplantation and Colonization

The *DAZL*-deficient rat line was produced on a Sprague Dawley background and expresses a small hairpin RNA (shRNA) transgene designed to stimulate degradation of transcripts encoding *DAZL* [17]. Rats in the *DAZL*-deficient transgenic line are male-sterile due to a defect in spermatogenesis; however, female rats from the same line remained fully fertile [17]. To prepare recipients for transplantation of spermatogonia, heterozygous *DAZL*-deficient male rats or their wildtype litter mates were injected (i.p.) with 12 mg/kg busulfan (4 mg/ml in 50% DMSO) at 12 days of age. Busulfan is an alkylating agent cytotoxic to spermatogonia used to increase the effectiveness of spermatogonial transplantations [31], [40], and clinically to combat cancer in humans [6], [22]. At 24 days of age (i.e. 12 days after busulfan treatment) donor *tgGCS-EGFP* rat spermatogonia harvested from culture at either passage 13 (culture day 158) or passage 17 (culture day 204) were loaded into injection needles fashioned from 100 µl glass capillary tubes at concentrations ranging from 2×10^3^ to 1.5×10^5^ EGFP^+^ cells/50 µl culture media containing 0.04% trypan blue. The entire 50 µl volume was then transferred into the seminiferous tubules of anesthetized rats by retrograde injection through the *rete* of their right testes [48]. The number of EGFP^+^ colonies formed/testis were scored using an Olympus IX70 fluorescence microscope (Olympus, Inc) to visualize donor cell transgene expression in the seminiferous tubules at 30 days following transplantation. Images of recipient testis were taken with a Nikon SMZ1500 fluorescence stereomicroscope.

### Morphometric Analysis of Rat Spermatogenic Cells

*Histological Analysis of Rat Testes*: Spermatogenesis was evaluated in hematoxylin and eosin (H&E) stained, 3 µm thick histological sections prepared from testes of wild-type and *DAZL*-deficient littermates at 4 months of age, and from *DAZL*-deficient recipients at 212 days post-transplantation with spermatogonia (i.e. ∼8 months of age). Prior to sectioning, the right testis of each animal was isolated, incubated overnight at 22–24°C in Bouin's fixative, washed thoroughly in 70% ethanol and then embedded in paraffin. The average numbers of Sertoli cells, gonocytes, type-A spermatogonia (Type-A), intermediate to type-B spermatogonia (Type-B), pre-leptotene spermatocytes (PL), leptotene to early pachytene spermatocytes (L-EP), mid-pachytene to diplotene spermatocytes (MP-D), round spermatids (RS) and elongating spermatids (ES) were scored/tubular cross section. Sections were prepared from triplicate animals for each genotype. Numbers of cells per tubular cross-section were counted for each of the above categories by morphometric analysis using the Simple PCI software (Simple-PCI, Inc.) in line with an AX70 light microscope (Olympus, Inc). Cells in cross sections of each rat were counted in microscopic fields of 15,400 µm^2^ (140 µm×110 µm) from at least 30 tubules/rat, n = 3 rats/strain. Average counts for each cell type were normalized per 1000 Sertoli cells from triplicate rats of each group. Spermatogenic cell types in wild-type rats were classified based on their morphologies in the H&E-stained sections and localization to specific stages of a seminiferous epithelial cycle [49]. The spermatogenic stage of each tubule used to count spermatogonia and Sertoli cells was verified in parallel cross-sections (3 µm) stained by the periodic acid-Schiff method in order to visualize steps of rat spermiogenesis. In *DAZL*-deficient rats, spermatogonia were classified as either undifferentiated (Undif) or differentiated (Dif) based on their patterns of H&E staining in comparison to wild-type rats. This was due to the inability to clearly identify distinct spermatogenic stages in the *DAZL*-deficient rats.

### Histological Analysis of Spermatogonial Types

In wild-type rats, the numbers of undifferentiated and differentiating spermatogonia per tubular cross section were first scored at stages VIII, XI, XIII, XIV, II and V of spermatogenesis to avoid scoring differentiating spermatogonia in M-phase. Undifferentiated spermatogonia were clearly distinguished from differentiating types during each stage due to their relative lack of nuclear staining. As bright field images, undifferentiated spermatogonia were lightly stained throughout the cytoplasm and nucleus, and appeared “pinkish” compared to differentiating types of spermatogonia. Between stages V and VII, nucleoli of A_al_ spermatogonia became more prominent as they differentiated into type A1 spermatogonia. In contrast, types A1 to B spermatogonia showed increasingly darker staining in distinct regions of their chromatin as they differentiated. Types A1 and A2 spermatogonia often showed “spots” of darkly stained chromatin situated randomly in the nucleus, which sharply contrasted from lighter staining chromatin throughout the rest of the nucleus. Nuclei of types A1 and A2 spermatogonia were clearly distinct from nuclei of undifferentiated spermatogonia. Types A3 to B spermatogonia also showed dark patterns of chromatin staining with increasing peri-nuclear localization of the most intensely stained regions.

### Fluorometric Analysis of EGFP in Rat Testes

Seminiferous tubules of recipient animals were dissected from the testes of 24 day old rats and then homogenized in 1.5 ml of ice-cold lysis buffer [50 mM HEPES pH 8.0, 150 mM NaCl, 1 mM EDTA, 10% glycerol, 1% TritonX-100, 10 µg/ml aprotinin, 10 µg/ml leupeptin and 1 protease inhibitor tablet/12.5 ml] for 30 seconds using a PTA-7 probe on setting 5 of a PT10-35 polytron (Kinematica). The homogenates were incubated on ice for 15–20 min and then centrifuged at 3000 x g for 10 min at 4°C in a GPR table-top centrifuge (Beckman, Inc.). The supernatant solutions were centrifuged at 15,800 x *g* for 10 min at 4°C in a micro-centrifuge (Model 5042, Eppendorf, Inc.), and the resultant supernatant fractions were then stored at −80°C. Frozen supernatant solutions were thawed on ice and then further clarified by centrifugation at 230,000 x *g*, r_av_ (tla-100.3 rotor, TL1000 ultracentrifuge, Beckman, Inc.) for 30 min at 4°C. Standards and supernatant solutions from the final centrifugation step were diluted into assay buffer [100 mM sodium bicarbonate, pH 9.6] and then analyzed for fluorescent intensity using a FL600 fluorescence micro-titer plate reader (BioTek, Inc.) equipped with filter wheel sets for maximal excitation at 485 nm and maximal emission at 516 nm. Affinity purified recombinant EGFP with a carboxyl-terminal histidine tag (rEGFP-His) was used as a standard for determining equivalents of EGFP in lysates prepared from testes of recipient animals. The rEGFP-His was produced by transient expression from the vector pcDNA6.0-EGFP-V5-His-B following transfection (Fugene 6 transfection reagent, Roche, Inc.) into COS-7 cells as previously described [48].

### Genotyping Progeny from Recipient Rats

Transgenic rat progeny from crosses between wild-type recipients and wild-type females were genotyped by PCR analysis of genomic DNA using primers specific to the *EGFP* transgene, and the *LTR* region of the lentiviral transgene used to produce the *DAZL*-deficient rat line; primers to *GAPDH* were used to amplify loading controls. Transgene copy number in F2 progeny from hemizygous crosses between F1 progeny of recipients and wildtype females was determined by qtPCR using primers to *EGFP* and the *18S* ribosomal subunit. Genotyping results were verified by Southern blot hybridization assays of genomic DNA digested with Xmn1 and Xba1 using a probe specific for the *EGFP* transgenes, and for olfactory marker protein as a loading control. The EGFP probe was isolated as a Nhe1/EcoR1 fragment from pLEGFP-C1 (Clonetech, Inc.). The OMP probe was isolated as a BamH1 fragment from pTOPO-XL:OMP corresponding to base pairs 14268243-14269267 of NW_047561; GI:34857865; RGSC v3.4 [31]. *EGFP* PCR primers: 5′-GTCTCGTGACCCTGACCTACGG-3′ and 5′-ATGCCCTTCAGCTCGATGCGG-3′; Rat *GAPDH* PCR primers: 5′-ATGATTCTACCCACGGCAAG-3′ and 5′-GCTAAGCAGTTGGTGGTGCA-3′; Lentiviral transgene PCR primers to *LTR* region: 5′-AACAGGGACTTGAAAGCG-3′ and 5′-ATACTGACGCTCTCGCACCC-3′. Genotyping results were also confirmed in representative progeny by direct visualization of *EGFP* transgene expression in testes and ovaries using a Nikon SMZ1500 fluorescence stereomicroscope.
